# Assessment of Postoperative Facial Nerve Outcomes Following Parotidectomy for Benign and Malignant Parotid Gland Lesions: A Prospective Observational Study

**DOI:** 10.7759/cureus.113950

**Published:** 2026-08-04

**Authors:** Riddhi Parmar, Riddhi Gohil, Sanjay Tota, Prerna Savaliya

**Affiliations:** 1 Otorhinolaryngology, Shantabaa Medical College and General Hospital, Amreli, IND

**Keywords:** facial nerve, facial nerve dysfunction, house-brackmann grading, marginal mandibular branch, parotidectomy, parotid neoplasms, salivary gland tumors

## Abstract

Background

Postoperative facial nerve dysfunction is the most feared complication of parotidectomy. This study determined its incidence, severity, branch-wise distribution, recovery pattern, and determinants, and compared the outcomes between benign and malignant parotid lesions.

Methods

In this prospective observational study, 120 consecutive adults underwent superficial, total conservative, or radical parotidectomy at a tertiary care hospital. Facial nerve function was graded using the House-Brackmann (HB) scale preoperatively and at defined postoperative intervals (postoperative day 1, one month, three months, and six months). Facial weakness persisting at six months was considered permanent for the purpose of this study. Outcomes were compared according to histopathology, extent of surgery, and tumor size using the chi-square test, Fisher's exact test, and the Cochran-Armitage trend test.

Results

The study included 120 patients with a mean age of 47.8±14.2 years; 66 were men and 54 were women. Of them, 82 (68.3%) had benign lesions, while 38 (31.7%) had malignant lesions. Facial nerve function remained normal in 72 (60.0%); 38 (31.7%) had temporary dysfunction, all recovering to HB grade I by six months, and 10 (8.3%) had permanent paralysis. The overall incidence of postoperative facial nerve dysfunction, including both temporary and permanent dysfunction, was higher in patients with malignant tumors than in those with benign tumors. Dysfunction occurred in 28 of 38 patients (73.7%) with malignant tumors and in 20 of 82 patients (24.3%) with benign tumors (relative risk, 3.02; 95% confidence interval, 1.97-4.62; p<0.001). The incidence of dysfunction also increased with the extent of surgery, from 20.3% after superficial parotidectomy to 58.1% after total conservative parotidectomy and 100% after radical parotidectomy (p for trend <0.001). Similarly, dysfunction increased with tumor size, from 8.6% for tumors <2 cm to 36.7% for tumors 2-4 cm and 92.0% for tumors >4 cm (p for trend <0.001). The marginal mandibular branch accounted for half of all deficits (24/48).

Conclusions

Facial nerve dysfunction after parotidectomy is common but predominantly transient. Malignant histology, greater resection extent, and larger tumor size were each strongly associated with postoperative facial nerve dysfunction. Risk-specific counseling, judicious selection of surgical extent, and early rehabilitation remain central to minimizing morbidity.

## Introduction

The parotid gland accounts for roughly 70% of salivary gland tumors, most of them benign. Approximately 75%-85% of parotid gland tumors are benign, whereas 15%-25% are malignant, although these proportions vary across populations [1,2]. A recent 10-year regional study from Bulgaria reported that 86.4% of parotid gland tumors were benign and 13.6% were malignant. Pleomorphic adenoma and Warthin tumor dominate among benign lesions, while mucoepidermoid and adenoid cystic carcinoma predominate among malignancies [3]. The facial nerve traverses the gland, dividing it into superficial and deep lobes before branching into temporal, zygomatic, buccal, marginal mandibular, and cervical divisions. This anatomy, compounded by tumor infiltration, fibrosis, and prior surgery, makes the nerve vulnerable whenever the gland is dissected.

Surgical excision ranging from extracapsular dissection or superficial parotidectomy to total conservative or radical parotidectomy remains the mainstay of treatment, chosen according to tumor size, lobe, and biology [4]. Despite advances in technique, magnification, and intraoperative monitoring, facial nerve dysfunction remains parotid surgery's most feared complication, with reported rates spanning an unusually wide range: 20% overall (17.2% transient, 4.5% permanent) in a 2,650-patient multicenter registry [5], as low as 5.2% in a predominantly benign, routinely monitored cohort [4], and as high as 47.9% in an unselected consecutive series [6]. Dysfunction may be transient, from traction, edema, thermal injury, or compression, or permanent, from transection, deliberate sacrifice, or perineural invasion, with consequences extending well beyond motor loss into ocular exposure, oral incompetence, dysarthria, and psychosocial distress [7,8]. The reported determinants include histology, tumor size and location, extent of resection, surgeon experience, and use of intraoperative monitoring [5,9,10].

Prospective data comparing facial nerve outcomes between benign and malignant parotid disease within a single cohort remain limited, particularly from South Asian centers where intraoperative monitoring is not universally available. This study was undertaken to determine the incidence, severity, branch-wise distribution, recovery pattern, and determinants of postoperative facial nerve dysfunction following parotidectomy, and to compare these outcomes between benign and malignant lesions.

## Materials and methods

Study design and setting

This prospective observational study was conducted in the Department of Otorhinolaryngology and Head and Neck Surgery, Narendra Modi Medical College and Hospital, Ahmedabad, Gujarat, India, between April 2025 and June 2026. Clinical, radiological, operative, and follow-up data were collected prospectively throughout the study period.

Ethical considerations

The study was conducted in accordance with the ethical principles of the Declaration of Helsinki. Approval was obtained from the Institutional Review Board of Narendra Modi Medical College, Ahmedabad (approval number NaMoMC/IRB/2025/211, dated March 28, 2025). Written informed consent was obtained from every participant after explanation of the nature and purpose of the study, the surgical procedure, potential complications, and follow-up requirements. Patient confidentiality and anonymity were maintained throughout.

Participants

A total of 120 consecutive patients with parotid gland lesions who underwent parotidectomy during the study period were enrolled after screening at the outpatient and inpatient departments. Consecutive sampling was employed, and all eligible patients undergoing parotidectomy during the study period were included. No formal sample size calculation was performed because this was a prospective observational study using consecutive sampling. All eligible patients undergoing parotidectomy during the predefined study period were enrolled to minimize selection bias and reflect routine clinical practice.

Inclusion criteria

The inclusion criteria for the study were: (a) age above 18 years; (b) a clinical/radiological/cytological diagnosis of a benign or malignant parotid lesion; (c) a plan for parotidectomy; and (d) willingness to provide informed consent and complete follow-up.

Exclusion criteria

The exclusion criteria were patients who (a) were medically unfit for surgery; (b) had pre-existing facial palsy unrelated to parotid disease; (c) had recurrent parotid disease previously treated elsewhere; (d) declined to provide informed consent or complete follow-up; and (e) had incomplete clinical records.

Preoperative evaluation

History and examination covered swelling duration, pain, facial weakness, mastication difficulty, skin change, cervical lymphadenopathy, tobacco/alcohol use, prior radiation, and comorbidity. Facial nerve function was assessed using the House-Brackmann (HB) grading system, a validated six-grade clinical scale for evaluating facial nerve function, ranging from grade I (normal facial function) to grade VI (complete facial paralysis) [11]. Ultrasonography was the initial imaging modality, with contrast-enhanced CT or MRI performed when deep lobe or adjacent-structure involvement needed clarification [12]. Fine needle aspiration cytology (FNAC) was performed in all patients, which guided preliminary diagnosis and surgical planning [13,14]. Routine preanesthetic work-up included complete blood count, liver and renal function tests, coagulation profile, blood glucose, chest radiography, and electrocardiography.

Surgical procedure

The extent of parotidectomy - superficial, total conservative, or radical - was determined preoperatively from clinical, radiological, and cytological findings. All procedures used a standard modified Blair incision under general anesthesia; the facial nerve trunk was identified by standard landmarks and traced distally along its branches. All procedures were performed by consultant otorhinolaryngologists experienced in parotid surgery, with resident assistance where appropriate. The maximum tumor diameter was recorded from the resected specimen and grouped a priori into three strata: <2.0 cm, 2.0-4.0 cm, and >4.0 cm. Excised specimens underwent histopathological examination, which established the final diagnosis. Intraoperative facial nerve monitoring was unavailable at our institution because of resource limitation; the facial nerve was identified and preserved by anatomical landmarks alone.

Postoperative assessment and follow-up

Facial nerve function was reassessed after anesthetic recovery, on postoperative day 1, at one week, and at one, three, and six months, using the HB scale and examined branch-wise (temporal, zygomatic, buccal, marginal mandibular, cervical). For the purpose of this study, facial weakness persisting at six months was considered permanent, consistent with the follow-up endpoint used in several contemporary parotidectomy outcome studies. Patients with facial weakness received eye protection, ocular lubrication, and facial physiotherapy [8]; those with malignant tumors were referred for adjuvant oncological management where indicated.

Statistical analysis

Data were analyzed in IBM SPSS Statistics Version 26 (IBM Corp., Armonk, NY, USA). Quantitative variables were expressed as mean±SD, categorical variables as frequencies and percentages. Categorical outcomes were compared with the Pearson chi-square test using Fisher's exact test (2x2) or the Fisher-Freeman-Halton exact test (larger tables) where the expected counts fell below five. The Cochran-Armitage test assessed dose-response trends across ordered categories of surgical extent and tumor size, and relative risks with 95% confidence intervals (CIs) were calculated for dysfunction by histological category. Two-sided p<0.05 was considered significant. This study is reported in accordance with the Strengthening the Reporting of Observational Studies in Epidemiology (STROBE) statement.

## Results

Demographic profile

All enrolled patients completed the scheduled six-month follow-up, and no patients were lost to follow-up. A total of 120 patients were analyzed: 66 men (55.0%) and 54 women (45.0%), a male-to-female ratio of 1.2:1. The mean age was 47.8±14.2 years (range 21-76 years), with most cases concentrated in the fourth and fifth decades. Mean maximum tumor diameter was 3.4±1.2 cm (range 1.5-6.8 cm) (Table 1).

**Table 1:** Demographic and tumor characteristics of patients undergoing parotidectomy (n=120) SD: Standard deviation, cm: centimeter.

Characteristic	Value
Age, mean±SD (years)	47.8±14.2
Age range (years)	21-76
Male, n (%)	66 (55.0)
Female, n (%)	54 (45.0)
Male-to-female ratio	1.2:1
Maximum tumor diameter, mean±SD (cm)	3.4±1.2
Tumor diameter range (cm)	1.5-6.8

Histopathological distribution

Benign lesions outnumbered malignant by more than two to one (82, 68.3% vs 38, 31.7%). Pleomorphic adenoma was commonest overall (52 cases, 43.3%), followed by Warthin tumor (18, 15.0%), basal cell adenoma (7, 5.8%), and oncocytoma (5, 4.2%). Among malignancies, mucoepidermoid carcinoma predominated (16, 13.3%), followed by adenoid cystic (nine, 7.5%), acinic cell (six, 5.0%), salivary duct (four, 3.3%), and carcinoma ex pleomorphic adenoma (three, 2.5%) (Table 2).

**Table 2:** Histopathological distribution of parotid gland lesions (n=120)

Histopathological diagnosis	Number of cases	Percentage of cohort (%)
Benign lesions	82	68.3
Pleomorphic adenoma	52	43.3
Warthin tumor	18	15
Basal cell adenoma	7	5.8
Oncocytoma	5	4.2
Malignant lesions	38	31.7
Mucoepidermoid carcinoma	16	13.3
Adenoid cystic carcinoma	9	7.5
Acinic cell carcinoma	6	5
Salivary duct carcinoma	4	3.3
Carcinoma ex pleomorphic adenoma	3	2.5
Total	120	100

Surgical procedures and overall facial nerve outcome

Superficial parotidectomy was the most common (74, 61.7%), followed by total conservative parotidectomy (31, 25.8%, mainly deep lobe or larger lesions) and radical parotidectomy with nerve sacrifice (15, 12.5%, exclusively for malignant tumors with nerve involvement) (Table 3). Facial nerve function remained normal in 72 patients (60.0%); temporary dysfunction occurred in 38 (31.7%, 95% CI 24.0-40.4%) and permanent paralysis in 10 (8.3%, 95% CI 4.6-14.7%), for an overall dysfunction rate of 40.0% (95% CI 31.7-48.9%). Every temporary case recovered to HB grade I within six months. This is shown in Table 3.

**Table 3:** Type of parotidectomy performed (n=120)

Type of procedure	Number of patients	Percentage (%)
Superficial parotidectomy	74	61.7
Total conservative parotidectomy	31	25.8
Radical parotidectomy	15	12.5
Total	120	100

Facial nerve outcome by histopathology

Malignant lesions carried roughly three times the risk of any dysfunction (73.7% vs 24.3%; relative risk 3.02, 95% CI 1.97-4.62; chi-square = 26.29, df=1, p<0.001) and roughly eight times the risk of permanent paralysis (21.1% vs 2.4%; relative risk 8.63, 95% CI 1.92-38.72; Fisher's exact p=0.002) compared with benign lesions (chi-square = 29.03, df = 2, p < 0.001 across all three categories) (Table 4).

**Table 4:** Postoperative facial nerve outcome by histopathological category

Facial nerve outcome	Benign (n=82), n (%)	Malignant (n=38), n (%)	Total (n=120)
Normal function	62 (75.6)	10 (26.3)	72
Temporary dysfunction	18 (22.0)	20 (52.6)	38
Permanent paralysis	2 (2.4)	8 (21.1)	10
Any dysfunction	20 (24.3)	28 (73.7)	48

Branch-wise facial nerve involvement

**Figure 1:**
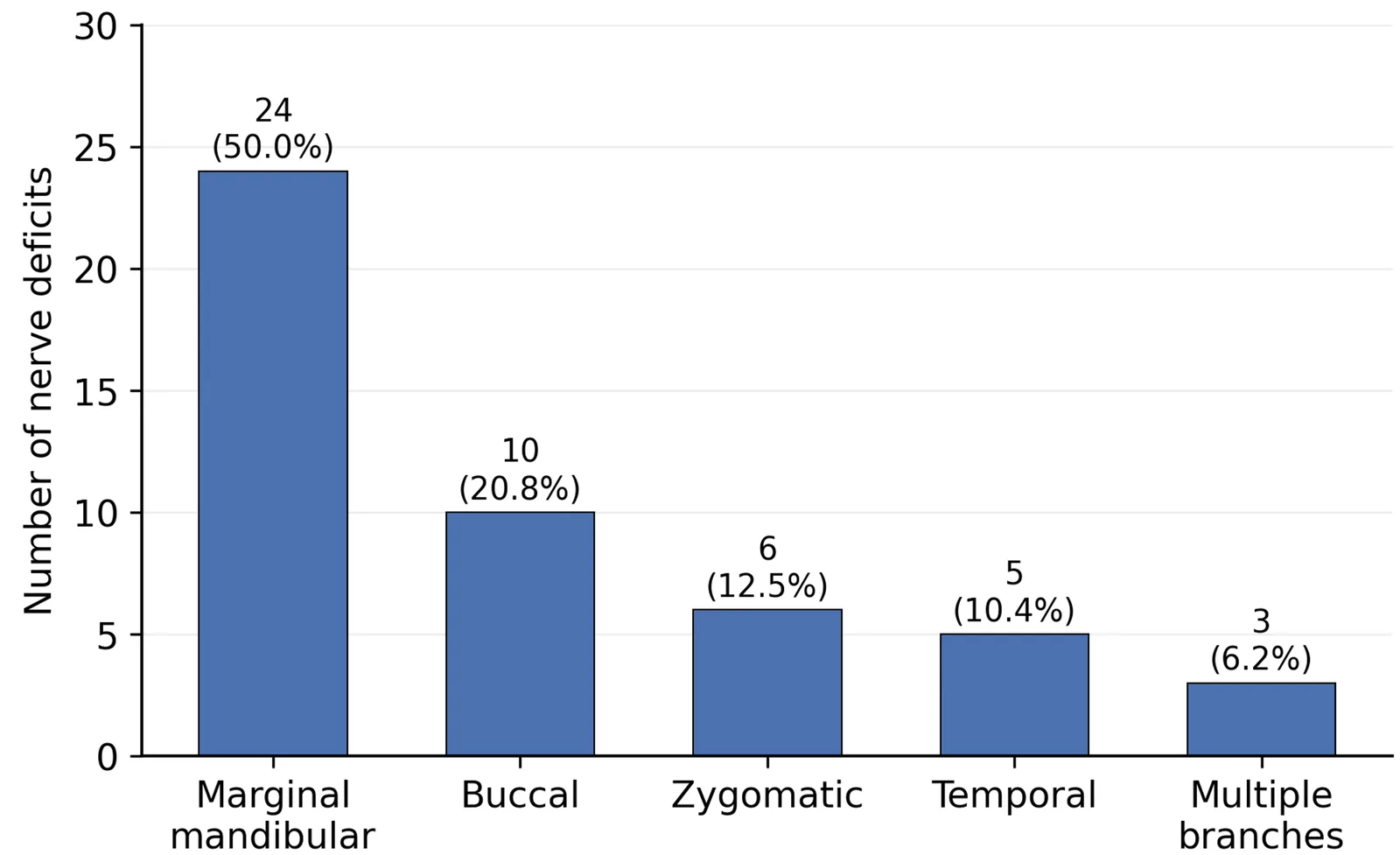
Branch-wise distribution of postoperative facial nerve deficits (n = 48).

Among the 48 deficits, the marginal mandibular branch alone accounted for half (24, 50.0%); buccal, zygomatic, and temporal branches together accounted for a further 43.7%, and 6.3% involved multiple branches in the context of extensive malignant disease. No isolated cervical branch deficit was observed (Figure 1).

Facial nerve outcome by extent of surgery and tumor size

**Figure 2:**
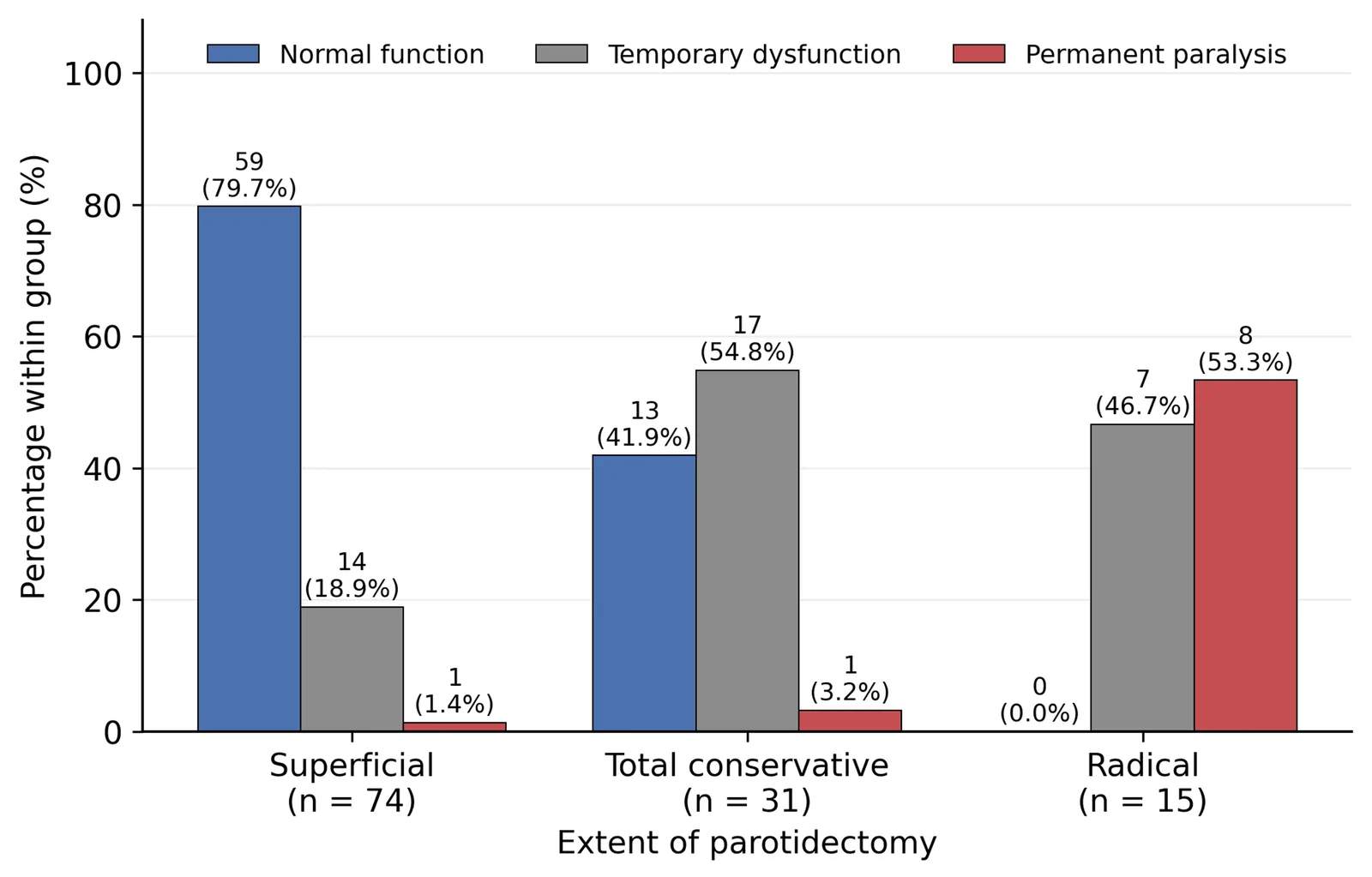
Postoperative facial nerve outcome according to the extent of parotidectomy Differences between surgical procedure groups were analyzed using the Fisher-Freeman-Halton exact test (χ²=67.36, df=4, p<0.001), and the ordered trend across increasing surgical extent was assessed using the Cochran-Armitage trend test (z=6.22, p<0.001).

**Figure 3:**
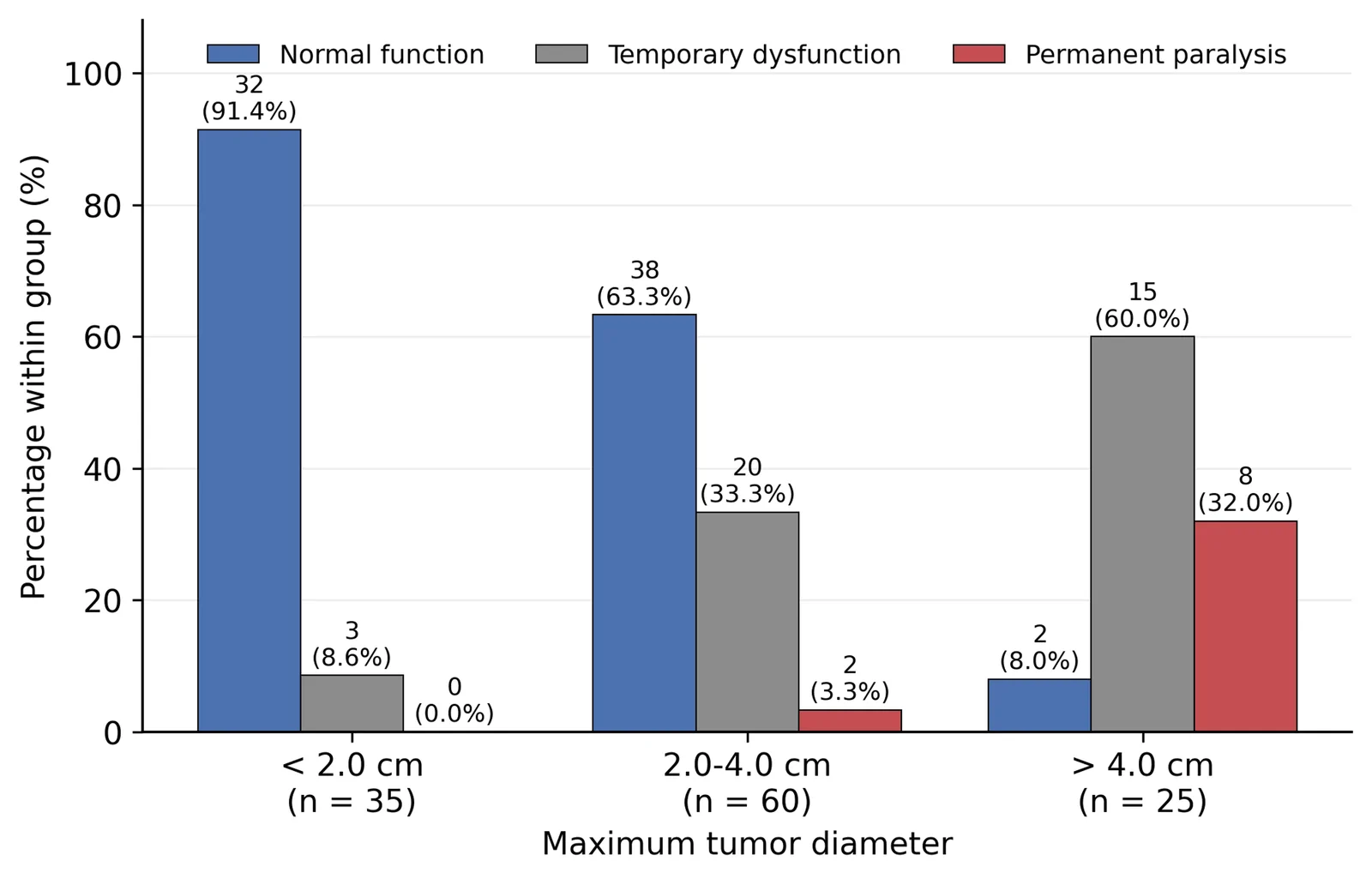
Postoperative facial nerve outcome according to maximum tumor diameter Differences in postoperative facial nerve outcomes among tumor size categories were analyzed using the Fisher-Freeman-Halton exact test (χ²=50.95, df=4, p<0.001). The ordered increase in postoperative facial nerve dysfunction with increasing tumor size was assessed using the Cochran-Armitage trend test (z=6.37, p<0.001).

The incidence of dysfunction increased progressively with the extent of resection - from 20.3% after superficial parotidectomy to 58.1% after total conservative parotidectomy and 100% after radical parotidectomy (Fisher-Freeman-Halton p<0.001; Cochran-Armitage trend z=6.22, p<0.001). Similarly, dysfunction increased with tumor size, from 8.6% for tumors <2.0 cm to 36.7% for tumors measuring 2.0-4.0 cm and 92.0% for tumors >4.0 cm (Fisher-Freeman-Halton p<0.001; Cochran-Armitage trend z=6.37, p<0.001). Permanent paralysis was almost confined to radical procedures (53.3% vs 1.9% of non-radical cases). The relationship between postoperative facial nerve dysfunction and both the extent of surgery and tumor size is illustrated in Figures 2, 3.

## Discussion

In this prospective series of 120 parotidectomies, temporary facial nerve dysfunction occurred in 31.7% of the patients and permanent paralysis in 8.3%. All temporary deficits resolved to HB grade I within six months. Malignant histology was strongly associated with both temporary and permanent dysfunction, and the marginal mandibular branch was the most frequently affected.

Comparison with published series

The reported dysfunction rates vary widely across the literature, driven mainly by case mix, surgical extent, use of intraoperative monitoring, and timing of assessment. Series restricted to benign disease report the lowest figures - 5.2% overall with routine monitoring [4], 3.75% with no total paralysis [15] - while registry-scale data occupy the middle ground (20% overall, 17.2% transient, 4.5% permanent, in 2,650 patients) [5], and unselected series including malignancies and radical procedures report considerably higher rates (up to 47.9-51.2%) [6,16]. Our rates of 31.7% temporary and 8.3% permanent dysfunction sit between these extremes, consistent with a cohort in which nearly one-third of patients had malignant disease and 12.5% underwent radical resection with deliberate nerve sacrifice. The timing of assessment also drives apparent incidence: Głuszkiewicz et al. found dysfunction in 74% of patients assessed at one month, but 0% permanent palsy by six months [17], indicating that early snapshots capture transient weakness that later resolves and should not be read as directly comparable to studies, including ours, that report a single cumulative rate through six months. The comparison is shown in Table 5.

**Table 5:** Comparison of postoperative facial nerve dysfunction with published series IFNM: intraoperative facial nerve monitoring. Rates are as reported in the source publications and are not directly comparable because of differences in case mix, surgical extent, use of IFNM, and the timing and definition of facial nerve assessment.

Study (year)	Design and case mix	n	Temporary dysfunction (%)	Permanent paralysis (%)
Cirignaco et al. (2025) [4]	Retrospective; benign only; routine IFNM	329	4.6	0.6
Mortensen and Bjørndal (2024) [15]	Retrospective; benign only	160	3.75 (overall palsy)	0
Warda et al. (2026) [5]	Multicenter registry; benign and malignant	2650	17.2	4.5
Salih et al. (2022) [18]	Retrospective; predominantly benign (85.8%)	127	9	1.6
Ruas et al. (2023) [6]	Retrospective; benign and malignant	48	47.9 (any dysfunction); 82.6% recovered	~8.3
Otanez et al. (2024) [16]	Retrospective; benign and malignant	78	32	19.2
Głuszkiewicz et al. (2022) [17]	Prospective; benign and malignant; assessed at 1 month	50	74.0 (at 1 month)	0 (at 6 months)
Present study	Prospective; benign and malignant	120	31.7	8.3

Determinants of outcome: histopathology, extent, and tumor size

The elevated risk in malignant disease has a clear biological basis: perineural invasion, dense peritumoral fibrosis, deep lobe extension, and adherence to surrounding structures make the nerve harder to separate from tumor and, in a subset of cases, leave sacrifice as the only route to clear margins. Malignancy, parapharyngeal extension, and pre-existing facial weakness each independently predicted complete paralysis in the largest registry to date [5], whereas a smaller cohort found that histology influenced the severity of dysfunction rather than its occurrence [6].

Where nerve sacrifice cannot be avoided, immediate reconstruction changes the calculus: reanimation performed in the same sitting as radical parotidectomy, by selective reinnervation or temporalis tendon transfer, restores usable movement in most patients [19], and adjuvant radiotherapy does not appear to undermine graft outcomes when reconstruction is planned upfront [20].

The gradients across the extent of surgery and tumor size observed here (Figures 2, 3) point to the same underlying biology: the incidence of dysfunction increased from one in five superficial parotidectomies to every radical case, mirroring prior multicenter data in which less extensive dissection was the strongest predictor of preserved early function [21,22], while dysfunction rose more than tenfold from the smallest to the largest tumor stratum, consistent with the largest available registry flagging tumors above 4 cm as a risk factor for more severe and prolonged dysfunction [5]. Because radical parotidectomy in this cohort was performed exclusively for malignant tumors with facial nerve involvement, surgical extent should not be interpreted as an independent causal predictor of postoperative dysfunction but rather as a surrogate marker of more advanced disease. Larger multicenter studies with multivariable adjustment are needed to distinguish the independent contributions of tumor biology and surgical extent.

Branch vulnerability

Half of all deficits here involved the marginal mandibular branch alone, consistent with its status as the branch most often injured in the largest available registry [5] and in a cohort where nearly three-quarters of patients had marginal mandibular weakness with the poorest recovery [7]. The anatomy explains this well: the branch runs superficially and largely unprotected across the inferior pole of the gland, often as a single trunk with little collateral supply, directly in the surgeon's working field.

Implications for practice

Three points follow. Preoperative counseling should reflect individual risk rather than a single generic warning, incorporating histological suspicion and imaging-derived tumor size where available. Intraoperative facial nerve monitoring warrants consideration where resources allow - a pooled analysis of over 1,000 patients found monitoring roughly halved the odds of immediate and permanent weakness [10], a benefit echoed elsewhere [4,23]. And because temporary deficits here recovered without exception, early eye protection and structured physiotherapy are worth instituting routinely [8]. Previous studies, including that by Yankov et al., have identified Frey's syndrome as one of the most frequent long-term sequelae after parotidectomy, particularly Frey's syndrome (gustatory sweating), which results from aberrant regeneration of parasympathetic fibers of the auriculotemporal nerve. Although this complication was not evaluated in the present study because of the six-month follow-up focused on facial nerve recovery, previous studies have identified Frey's syndrome as one of the most frequent long-term sequelae after parotid surgery, with the potential to adversely affect patient comfort and quality of life. Its recognition underscores the importance of long-term follow-up and patient counseling regarding delayed postoperative complications [24]. It is also worth noting that facial weakness is not necessarily what patients remember most: longer-term follow-up after benign parotidectomy repeatedly finds great auricular nerve numbness, not facial palsy, rated as most troublesome [25,26] - a dimension this study's six-month window was not designed to capture, but one that should shape future outcome measurement.

Limitations

This single-center study has several limitations. As a tertiary referral center, the institution may receive a relatively greater proportion of complex and malignant parotid tumors than primary or secondary care hospitals, potentially limiting the generalizability of these findings. Intraoperative facial nerve monitoring was not available and was not used, so its potentially protective effect on both immediate and permanent weakness [10] could not be assessed here, and our rates may not be directly comparable with centers where monitoring is routine. The House-Brackmann scale is relatively insensitive to isolated marginal mandibular weakness; branch-specific instruments such as the modified Sunnybrook system may have detected additional deficits [7]. Six-month follow-up is conventional but may miss late recovery. Some patients may continue to demonstrate neurological recovery beyond six months; therefore, longer follow-up could provide a more accurate estimate of permanent facial nerve dysfunction. The malignant subgroup (n=38) limited the statistical power for adjusted analyses. Although histopathology, tumor size, and extent of surgery were all associated with postoperative facial nerve dysfunction, these variables were strongly interrelated because larger malignant tumors were more likely to undergo radical resection. Consequently, multivariable logistic regression was not performed, as the limited number of outcome events and substantial collinearity would have produced unstable estimates. Therefore, the associations reported should be interpreted as unadjusted findings. Another limitation of this study is that facial nerve dysfunction persisting at six months was classified as permanent. Although most recovery occurs within this period, some patients may experience delayed functional improvement beyond six months, which could have resulted in an overestimation of the rate of permanent facial nerve dysfunction.

## Conclusions

Postoperative facial nerve dysfunction after parotidectomy is common but is predominantly transient, with most patients recovering during follow-up. Malignant histology, more extensive surgery, and larger tumor size were associated with an increased risk of postoperative facial nerve dysfunction. These findings support individualized preoperative risk counseling, careful surgical planning, and early postoperative rehabilitation to minimize morbidity. Meticulous nerve dissection, selection of the least extensive procedure consistent with oncological adequacy, risk-specific preoperative counseling, and early eye protection and physiotherapy remain the cornerstones of minimizing facial nerve morbidity. Intraoperative monitoring and immediate reconstruction where nerve sacrifice is unavoidable merit consideration where not yet routine.
